# Local domain generalization with low-rank constraint for EEG-based emotion recognition

**DOI:** 10.3389/fnins.2023.1213099

**Published:** 2023-11-07

**Authors:** Jianwen Tao, Yufang Dan, Di Zhou

**Affiliations:** ^1^Institute of Artificial Intelligence Application, Ningbo Polytechnic, Zhejiang, China; ^2^Industrial Technological Institute of Intelligent Manufacturing, Sichuan University of Arts and Science, Dazhou, China

**Keywords:** domain adaptation, subdomain generalization, emotion recognition, electroencephalogram, local learning

## Abstract

As an important branch in the field of affective computing, emotion recognition based on electroencephalography (EEG) faces a long-standing challenge due to individual diversities. To conquer this challenge, domain adaptation (DA) or domain generalization (i.e., DA without target domain in the training stage) techniques have been introduced into EEG-based emotion recognition to eliminate the distribution discrepancy between different subjects. The preceding DA or domain generalization (DG) methods mainly focus on aligning the global distribution shift between source and target domains, yet without considering the correlations between the subdomains within the source domain and the target domain of interest. Since the ignorance of the fine-grained distribution information in the source may still bind the DG expectation on EEG datasets with multimodal structures, multiple patches (or subdomains) should be reconstructed from the source domain, on which multi-classifiers could be learned collaboratively. It is expected that accurately aligning relevant subdomains by excavating multiple distribution patterns within the source domain could further boost the learning performance of DG/DA. Therefore, we propose in this work a novel DG method for EEG-based emotion recognition, i.e., Local Domain Generalization with low-rank constraint (LDG). Specifically, the source domain is firstly partitioned into multiple local domains, each of which contains only one positive sample and its positive neighbors and *k*_2_ negative neighbors. Multiple subject-invariant classifiers on different subdomains are then co-learned in a unified framework by minimizing local regression loss with low-rank regularization for considering the shared knowledge among local domains. In the inference stage, the learned local classifiers are discriminatively selected according to their importance of adaptation. Extensive experiments are conducted on two benchmark databases (DEAP and SEED) under two cross-validation evaluation protocols, i.e., cross-subject within-dataset and cross-dataset within-session. The experimental results under the 5-fold cross-validation demonstrate the superiority of the proposed method compared with several state-of-the-art methods.

## Introduction

In the field of affective computing research (Mühl et al., 2014), automatic emotion recognition (AER; Dolan, 2002) has received considerable attention from computer vision communities (Kim et al., 2013). Many EEG-based emotion recognition methods have been proposed so far (Musha et al., 1997; Jenke et al., 2014; Zheng, 2017; Niu et al., 2018; Pandey and Seeja, 2019; Chang et al., 2021, 2023; Zhou et al., 2022). From the viewpoint of machine learning, EEG-based AER can be modeled as a classification or regression problem (Kim et al., 2013; Zhang et al., 2017), in which state-of-the-arts for AER usually tailor their classifiers trained on multiple subjects and apply them to individual subjects. From both qualitative and empirical observations, the generalizability of AER could be attenuated partly due to the individual differences among subjects (Jayaram et al., 2016; Zheng and Lu, 2016; Lan et al., 2018). That is, the subject-independent classifier usually achieves an inferior generalization performance since emotion patterns may significantly vary from one subject to another (Pandey and Seeja, 2019). As a possible solution, subject-specific classifiers are usually impractical due to insufficient training data (Li X. et al., 2018; Zhou et al., 2022). While conspicuous progress has been made to conquer this issue by improving feature representations and learning models (Zheng and Lu, 2015; Song et al., 2018; Li et al., 2018a,b; Li Y. et al., 2019; Du et al., 2020; Zhong P. et al., 2020; Zhou et al., 2022), there still exists a long-standing challenge incurred by individual diversities in EEG-based AER. This challenge is primarily attributed to the fact that the learned classifiers should be generalized into previously unseen subjects that may obviously differ from those on which the classifiers are trained (Ghifary et al., 2017). To this end, numerous domain adaptation (DA) learning algorithms for AER have emerged by exploiting EEG features (Zheng et al., 2015; Chai et al., 2017; Li J. et al., 2019; Pandey and Seeja, 2019; Zhang et al., 2019b; Li et al., 2020; Chen et al., 2021; Dan et al., 2021; Tao et al., 2022). For instance, Pandey and Seeja (2019)) and Li X. et al. (2018) successively proposed two subject invariant models for EEG-based emotion recognition; following the deep network architecture, in the researchers (Chai et al., 2016; Li H. et al., 2018; Luo et al., 2018; Li et al., 2018c, 2021; Wang et al., 2022; Zhou et al., 2022) designed several deep learning models for EEG-based emotion recognition.

Unfortunately, in some practical AER applications, the whole target data of interest may be unavailable in the stage of training a subject-specific classifier (Wang et al., 2022). In this case, domain generalization (DG; Muandet et al., 2013), an effective variant of DA (Bruzzone and Marconcini, 2010), is proved to be a feasible solution for DA emotion recognition (Tao et al., 2022). With no need to focus on the generalization of some specific target domain, DG methodology could better acquire out-of-the-distribution effects on test samples from other previously unseen target domains (Wang et al., 2022). While DA and DG are closely related in learning scenarios, DA algorithms generally cannot be directly applicable to DG since they rely on the availability of the target domain in the stage of training. In this sense, DG is more challenging than DA as no target data can be used for fine-tuning in the training stage (Ghifary et al., 2017).

**Figure 1:**
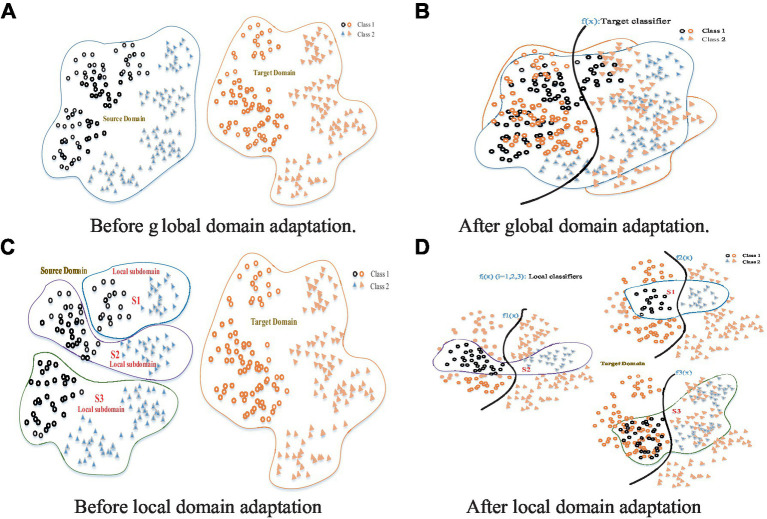
Global domain adaptation might lose some fine-grained information **(A,B)**. Local domain adaptation can exploit the local discriminative structures to capture the fine-grained information for each category **(C,D)**.

In DA/DG, one major problem is how to reduce or eliminate the distribution discrepancy between different domains (Patel et al., 2015; Wang et al., 2022). First of all, one needs to design a robust and effective criterion that can measure the domain discrepancy. Due to its simplicity, effectiveness, and intuition, Maximum Mean Discrepancy (MMD; Gretton et al., 2009) is a commonly adopted distribution distance measure criterion. Preceding MMD-based DA methods (Pan et al., 2011; Duan et al., 2012; Tao et al., 2012, 2017, 2019; Chen et al., 2013; Long et al., 2014a; Ding et al., 2018a,b,c), however, generally focused on the global statistical distribution shift between/among different domains without considering the complementarities and diversities between two subdomains constructed with local structures within the same/different domains (Gao et al., 2015; Zhu et al., 2020). This could result in attenuated adaptation performance to some extent, since not only could all the samples from both source and target domains be confused together, but also the local discriminative structures could be trimmed without capturing the fine-grained local structures (Zhu et al., 2020). That is, while the global distribution alignment may lead to approximate zero distribution distance between different domains, a common challenge that exists in preceding global methods is that the samples from different domains are pulled too close to be accurately classified. An intuitive example is shown in Figure 1, where the source domain presents a certain multimodal structure (as shown in Figure 1A). After global domain adaptation, as shown in Figure 1B, the distributions of the two domains are approximately the same, but the data in different semantic structures are too close to be classified accurately. This is a common problem in previous global DA methods. Hence, matching the global source and target domains may not work well in this scenario.

Concerning the challenge of global domain shift, several works pay attention to semantic alignment or matching conditional distribution (Long et al., 2014a, 2017). There are other works proposed to discover multiple latent domains by decomposing the source domain (Judy et al., 2012; Gao et al., 2015). While they have presented the effectiveness of DA by exploring multiple subdomains potentially existing in the source domain, discovering multiple representative latent domains is still a non-trivial task by explicitly dividing the source samples into multiple blobs (Zhu et al., 2020). Further, to overcome the shortages that exist in the global distribution measure, numerous deep subdomain adaptation methods have focused on accurately aligning the distributions between different subdomains (Gao et al., 2015; Zhu et al., 2020). For instance, the recent work in Zhu et al. (2020) focuses on aligning the distribution of the relevant subdomains within the same category in the source and target domains. These deep learning methods, however, usually contain several updatable loss functions and converge slowly. Moreover, it is still an unexplained open problem whether the success of deep DA methods really benefits from the feature representations, fine-tuned classifiers, or effects of the adaptation regularizers (Tao et al., 2022).

Motivated by the idea of subdomain adaptation, we propose in this work a Local Domain Generalization (LDG) scheme to implicitly align the relevant local domain distributions from a single source with that of the target domain. A key improvement of LDG over previous DG/DA methods is the capability of the fine-grained alignment of a domain shift by capturing the local discriminative structures in the source domain by excavating multiple subdomains as per each positive sample with its two k-NN subsets (as shown in Figure 1C). In these local domains, multiple classifiers can be jointly trained in a unified framework by aligning them with a referenced model. Under this framework, the model discrepancies between the relevant subdomains from the source and the target domain could be measured by considering the weights as per different distribution distances. After local domain adaptation, as shown in Figure 1D, each local domain distribution from the source domain is approximately the same as that of the target domain. Therefore, multiple local classifiers jointly learned with these local domain adaptations could be integrated and generalized into the target domain.

Specifically, we present an LDG framework for AER with EEG features with low-rank constraints. Under this framework, the source domain is firstly divided into multiple local domains, each containing only one positive sample (or exemplar; Zhang et al., 2016) and its positive and *k*_2_ negative neighbors. Intuitively, the distribution structures of these local domains for those exemplars are expected to be relatively closer and simpler than that of the global one. In LDG, multiple subject-invariant classifiers on different local domains are co-learned in a joint framework by minimizing local regression loss. Instead of evaluating the importance of each classifier individually, LDG selects models in a collaborated mode by considering the shared knowledge among local domains by additionally imposing a nuclear-norm-based regularizer on the objective function. The learned local classifiers are discriminatively selected according to their weights in the inference stage. While the DG performance of LDG also can be boosted with most feedforward network models by exploiting the deep feature representations, it does not need iterative deep training and converges fast, thus being very efficient and effective.

Different from the existing DG methods that only focus on global distribution alignment in the source domain(s), we consider the local distribution structures of the source domain and their relevance with the target domain to further enhance the effectiveness and generalizability of the learned adaptation model. Our algorithm can adapt as much knowledge as possible from a certain source domain, even if the EEG features between domains are partially distinct but overlapping. To the best of our knowledge, there is no prior work imposing DG with multiple local domains on solving AER problems. The main contributions of this paper are summarized as follows.

1. We propose a local domain generalization framework (LDG) for EEG-based emotion recognition by leveraging multiple structure-similar local domains from the source domain with multi-model distribution patterns. Using this framework, the capacity of MMD-based DA methods can be extended by excavating the local discriminative structures for each domain by aligning KNN-based local domain distributions.
2. We present a subdomain division strategy, i.e., splitting the source domain into multiple local domains, each of which is composed of each positive (exemplar) sample (Zhang et al., 2016; Li W. et al., 2018; Niu et al., 2018) and its *k*_1_ positive and *k*_2_ negative neighbors. Multiple local classifiers can be, respectively, trained on each local domain. We then formulate a new objective function by imposing a nuclear-norm-based regularizer on the model matrix in the objective function to further enhance the discriminative capability of the learned local classifiers by exploiting the intrinsic discriminative structure in the source domain.
3. An iterative optimization algorithm is presented for solving the objective of LDG that can be applied to EEG-based AER problems. The convergence of the optimization procedure can be guaranteed in terms of the proof of the proposed convergence theorem.
4. Extensive experiments are conducted on two benchmark databases (DEAP and SEED) under two cross-validation evaluation protocols (cross-subject within-dataset and cross-dataset within-session). The remarkable experimental results show that our method outperforms other state-of-the-art methods on emotion recognition tasks.

The rest of the paper is organized as follows. Section 2 reviews several related works in emotion recognition, DG, and subdomain adaptation. Section 3 introduces our LDG framework including the overall objective function, and then the optimization algorithm and its convergence analysis are successively provided in Section 4. Section 5 provides a series of experiments to evaluate the effectiveness of LDG for AER. Finally, we summarize the entire paper in Section 6.

## Related work

In recent decades, increasing attention has been given to emotion recognition with brain-computer interfaces (BCI; Dolan, 2002; Kim et al., 2013; Mühl et al., 2014) in the affective computing community. A vanilla aBCI system using spontaneous EEG signals firstly extracts sufficient discriminative features from the EEG data by a certain feature extractor and then trains an optimal classifier using these features and the corresponding emotion states for AER. Therefore, a proper design of EEG-based emotion recognition models helps facilitate the data processing, benefits from discriminant feature characterization, and lightens the model performance. The latest works about affective BCI (aBCI) usually adopt machine learning algorithms on automatic emotion recognition (AER) using extracted discriminative features (Musha et al., 1997; Jenke et al., 2014; Chang et al., 2023). However, the traditional machine learning method has a major disadvantage in that the feature extraction process is usually cumbersome, and relies heavily on human experts. Then, end-to-end deep learning methods emerged as an effective way to address this disadvantage with the help of raw EEG signals and time-frequency spectrums (Han et al., 2022). More details can be found in Zhang et al. (2020c), which investigated the application of several deep learning models to the research field of EEG-based emotion recognition, including deep neural networks (DNN) (Chang et al., 2021), convolutional neural networks (CNN), long short-term memory (LSTM), and a hybrid model of CNN and LSTM (CNN-LSTM; Zhong Q. et al., 2020; Mughal et al., 2022; Xu et al., 2022).

While preceding methods have obtained remarkable achievements on EEG-based AER (Zheng, 2017; Li et al., 2018a,b; Li Y. et al., 2019; Pandey and Seeja, 2019), the performance expectation for cross-subject/dataset recognition could be lowered due to the diversities of emotional states among subjects/datasets (Jayaram et al., 2016; Zheng and Lu, 2016; Li X. et al., 2018). While subject-specific classifiers may be a possible solution for these cases, they are usually infeasible in real tasks due to insufficient training data. Moreover, even if they are feasible in some specific scenarios, it is also an indispensable task to fine-tune the classifier to maintain a sound recognition capacity partly because the EEG signals of the same subject sometimes change (Zhou et al., 2022). Fortunately, the recently proposed domain adaptation (DA) technique (Patel et al., 2015) can be leveraged to surmount these challenges for EEG-based emotion recognition. As a well-focused research direction, the unsupervised domain adaptation (UDA) methodology has promoted a large amount of research effort devoted to generalizing the knowledge learned from one/multiple labeled source domain(s) into a different but related unlabeled target domain (Wang and Mahadevan, 2011; Gong et al., 2012; Long et al., 2014b, 2015, 2016; Ganin and Lempitsky, 2015; Ganin et al., 2016; Judy et al., 2017; Tzeng et al., 2017; Ding et al., 2018a,b,c). Over the past decade, DA-based emotion recognition methods have been a hot topic (Lan et al., 2018), almost fully covered in the literature of aBCI (Zheng et al., 2015; Chai et al., 2016, 2017; Jayaram et al., 2016; Zheng and Lu, 2016; Li H. et al., 2018; Li X. et al., 2018; Luo et al., 2018; Li et al., 2018c, 2020, 2021; Li J. et al., 2019; Chen et al., 2021; Dan et al., 2021; Tao et al., 2022; Zhou et al., 2022). Existing methods explore tackling different challenges in AER with EEG datasets by excavating a certain latent subspace shared by different domains for filling the domain distance among subjects or sessions.

In some real DA-based AER applications, the whole target data of interest may be unavailable in the stage of training (Ghifary et al., 2017). In this scenario, domain generalization (DG; Muandet et al., 2013), an effective variant of DA, has been proven to be a feasible solution for DA emotion recognition since it need not focus on the generalization of a certain specific target domain. While DA and DG are closely related in learning scenarios, DA algorithms generally are not directly applicable to DG since they rely on the availability of the target domain in the stage of training. In this sense, DG is more challenging than DA as no target data can be used for fine-tuning in the training stage. The extant works about DG can be divided into two research lines in terms of different strategies, i.e., feature-centric DG (Judy et al., 2012; Muandet et al., 2013; Ghifary et al., 2017; Motiian et al., 2017) and classifier-centric DG (Xu et al., 2014; Ghifary et al., 2015; Niu et al., 2015, 2018; Gan et al., 2016; Li W. et al., 2018). The former aims to mine domain-invariant features, while the latter uses multi-classifiers adaptation by regulating their weights. More research progress on DG can be found in the recent survey on DG (Wang et al., 2022).

As is known, a major task in vanilla UDA/DG methodology is to mitigate the domain discrepancy either by aligning the statistical moments (Pan et al., 2011; Duan et al., 2012; Tao et al., 2012; Chen et al., 2013; Long et al., 2014a,b; Xiao and Guo, 2015; Ding et al., 2018a,b,c) or by using domain adversarial learning (Ganin and Lempitsky, 2015; Ganin et al., 2016; Tzeng et al., 2017; Long et al., 2018; Pei et al., 2018) benefited from the powerful deep neural networks. Traditional DA/DG methods usually assume a global distribution shift between different domains and expect approximately the same global distribution of two domains after adaptation (Mansour et al., 2009). However, most of the preceding DA/DG methods face a common problem in that they only pay attention to matching the global statistical distribution between domains without considering the complementarities and diversities among subdomains constructed using several local structures within the same/different domains (Zhu et al., 2020). This could result in attenuated adaptation performance in part because the samples from different domains are pulled too close to be accurately classified in those global methods. As a result, not only will all the data from the source and target domains be confused, but also the discriminative structures can be mixed up. Subdomain adaptation can to some extent conquer the shortcomings in aligning global domain discrepancy. For instance, several related works have been proposed to excavate multiple latent domains from the source domain (Judy et al., 2012). To discover multiple representative latent domains, however, is a non-trivial task done by explicitly dividing the source samples into multiple blobs. Aiming at the disadvantages of global domain adaptation, considerable works (Gao et al., 2015; Zhu et al., 2020) have explored subdomain adaptation, which focuses on aligning the local domain discrepancies. Most deep DA/DG methods belong to the deep adversarial learning methodology and converge slowly due to several loss functions. To this end, Zhu et al. (2020) recently presented a deep subdomain adaptation network (DSAN) based on the proposed local maximum mean discrepancy (LMMD), which learns a DA network by aligning the related distributions of subdomains across different domains.

It is worth noting that the discriminative structures could still be mixed up in extant subdomain adaptation schemes when the source (or target) domain presents a multimodal distribution structure (as shown in Figure 1). Different from these works on aligning global/sub-domain(s) shift(s), we propose a novel fine-grained DG method for EEG-based emotion recognition, in which multiple patches (local domains) are firstly reconstructed from the source dataset and multiple local classifiers are then learned collaboratively for effective generalization into the target domain even with multiple kinds of distribution pattern (Gao et al., 2015). Our method does not need deep training and converges fast, while its adaptation expectation can be easily boosted with deep feature representations from most feedforward network models.

## Proposed framework

### Preliminary notations

In the context of this paper, we, respectively, denote by small and capital letters the column vectors and matrices. The frequently used notations are summarized in Table 1. The concatenation operations of matrices along the row (horizontally) are denoted as $\left[A_{1},\;A_{2},\ldots ,\;A_{k}\right]$, and their concatenation along the column (vertically) are denoted as $\left[A_{1},\;A_{2},\ldots ,\;A_{k}\right]$.

**Table 1:** Notations and descriptions.

Notations	Descriptions
*n*	Data size.
*d*	Feature dimensionality of data.
χ	Data space.
Γ	Label space.
$a={\left[a_{1},\;a_{2},\ldots ,\;a_{d}\right]}^{T}\in {\mathbb{R}}^{d}$	Feature vector.
$A\in {\mathbb{R}}^{n\times d}$	Data matrix.
*A*_*i*,*j*_	The (*i*, *j*) entry of *A*.
*A*_*i*_ and *A*_*j*_	The *i*-th row and *j*-th column of *A*.
*A^T^* and *a^T^*	The transpose of matrix *A* and vector *a*.
*tr*(*A*)	The trace of a matrix *A*.
$\langle A_{1},A_{2}\rangle =tr\left(A_{1}^{T}A_{2}\right)$	The inner product of two matrices *A*_1_ and *A*_2_.
${\left\|a\right\|}_{p}={\left(\sum_{i=1}^{d}{\left|a_{i}\right|}^{p}\right)}^{1/p}$	The *p*-norm of a vector *a*.
${\left\|A\right\|}_{F}=\sqrt{\sum_{i=1}^{n}\sum_{j=1}^{d}A_{i,j}^{2}}$	The Frobenius norm of *A*.
*I_r_*	Identity matrix of size *r* × *r*.
**1** *_d_*	d-dimensional vector of ones.
**0**_d_	d-dimensional vector of zeroes.

**Definition 1** (Local domain): For a certain domain $X={\left\{x_{i}\right\}}_{i=1}^{m}$ with some probability distribution P, a local domain for one positive example $x_{v}\in X$ is composed of its *k*_1_ positive nearest neighbor set $N_{k_{1}}^{+}\left(x_{v}\right)=\left\{x_{v_{1}},\ldots ,x_{v_{k_{1}}}\right\}$ and *k*_2_ negative neighbor set $N_{k_{2}}^{-}\left(x_{v}\right)=\left\{x_{v_{k_{1}+1}},\ldots ,\;x_{v_{k_{1}+k_{2}}}\right\}$, i.e.,$X_{v}=\left\{x_{v},N_{k_{1}}^{+}\left(x_{v}\right),N_{k_{2}}^{-}\left(x_{v}\right)\right\}$.

According to Definition 1, for any source domain $X^{s}={\left\{x_{i}^{s}\right\}}_{i=1}^{n_{s}}$ with *p* positive samples ${\left\{x_{v}^{s}\in {\mathbb{R}}^{d}\right\}}_{v=1}^{p}$ and *n_s_* – *p* negative samples, one can reconstruct *p* local domains $X_{v}^{s}=\left\{x_{v}^{s},N_{k_{1}}^{+}\left(x_{v}^{s}\right),N_{k_{2}}^{-}\left(x_{v}^{s}\right)\right\}$, 1≤v≤p, by finding the positive nearest neighbor set $N_{k_{1}}^{+}\left(x_{v}^{s}\right)=\left\{x_{v_{1}}^{s},\ldots ,x_{v_{k_{1}}}^{s}\right\}$ and *k*_2_ negative neighbor set for each positive sample $x_{v}^{s}$ (1≤v≤p).

**Definition 2** (Local domain adaptation, LDA): Let $\Delta =\left\{X_{1}^{s},\ldots ,X_{m}^{s}\right\}$ be a set of *m* local domains and $X^{t}\notin \Delta$ be a target domain. The task of LDA is to learn an ensemble function $f_{X^{t}}:X\to \Gamma$ by co-learning multiple classifiers $f_{v}\left(X_{v}^{s}\right)$ (1≤v≤m) given Δ and *X^t^* as the training examples by alleviating the distribution difference between source and target domains.

**Definition 3** (Local domain generalization, LDG): In this scenario, the target domain is inaccessible in the training stage. Given *m* local domains $\Delta =\left\{X_{1}^{s},\ldots ,X_{m}^{s}\right\}$, and denoted by $X_{a}^{s}={\left\{x_{i}^{a},y_{i}^{a}\right\}}_{i=1}^{n_{a}}$ the samples drawn from the *a*-th subdomain, the task of LDG is to co-learn multiple adaptive functions $f_{X_{a}^{s}}:X\to \Gamma \;$ only given $X_{a}^{s},\forall a=1,\ldots ,m$ as the training examples, which could be well-generalized to a certain unseen target domain.

### Motivation

As is known, a major task in vanilla UDA/DG methodology is to diminish the domain discrepancy either by aligning the statistical moments (Koelstra et al., 2012; Gao et al., 2015; Li et al., 2018a, 2020) or by domain adversarial learning (Gong et al., 2012; Lan et al., 2018; Li X. et al., 2018; Ding et al., 2018a) benefited from the powerful deep neural networks (Zhu et al., 2020; Zhou et al., 2022). While extensive exploration of cross-subject/session has been conducted effectively in the prior works by leveraging various domain adaptation tricks, one obvious shortage in these works is they usually assume a global distribution shift between different subjects and expect an approximately similar global distribution of two subjects after adaptation. In other words, these DA-based AER methods only focus on matching the global statistical distribution between subjects without considering the complementarities and diversities among local domains constructed using some intrinsic structures within the same/different subjects. This leads to attenuated adaptation performance since the real-world EEG data is usually quite diverse and the distribution of emotion data is complex. It is challenging to reduce the global distribution discrepancy between different domains.

As far as we know, limited effort, however, has been witnessed in improving DA/DG performance by leveraging local knowledge among multiple subdomains from a single source. The ignorance of the fine-grained local discriminative structures may result in unsatisfying generalization capacity in DA/DG. Exploiting the relationships among multiple local domains to match their distribution divergences could not only align the global statistical distributions but also the local discriminative patterns. In many real applications, the local structure is more important than the global structure (Ding et al., 2018a), and the local learning algorithms often outperform global learning algorithms (Ding et al., 2018b). Because of this, LDA/LDG is able to compensate for the limitation of global DA since the diversities of domain distributions intrinsically exist in real applications.

Motivated by this idea, we propose in this paper a novel domain generalization framework for EEG-based emotion recognition, i.e., Local Domain Generalization (LDG) with low-rank constraints. Under this framework, LDA is a relaxed extension of LDG, where the target domain of interest is provided during the training process. Specifically, the source domain of the auxiliary is firstly partitioned into multiple local domains, each of which contains only one positive sample (or called exemplar sample) and its *k*_1_ positive neighbors and *k*_2_ negative neighbors. Each local domain is expected to be relatively more similar and possess a simpler distribution structure. Then multiple subject-invariant local classifiers are co-learned on these local domains by minimizing a unified local regression loss. Instead of evaluating the importance of each classification model individually, LDG selects models in a collaborated mode for considering the shared knowledge among local domains by additionally introducing a nuclear-norm-based regularizer into the objective function. In the inference stage, the learned local classifiers are discriminatively selected and reweighted according to the distribution distance between each local domain and the target domain of interest.

In the following sections, we will present the objective formulation of our framework followed by its effective optimization algorithm.

### General formulation

In LDA/LDG learning, however, there still exists two challenges worthy to be effectively addressed: (1) how to divide one source into multiple local domains and (2) how to compute the weight of each sample in its local domain. Until now, little research has been reported to address these challenges for EEG-based emotion recognition through local regression learning by decomposing the source domain into multiple local domains. To address these challenges, in this section, we propose the general formulation of our framework underpinned by the robust local regression principle and the regularization theory. Concretely, our proposed method will possess several complementary characters, which can be combined into one unified optimization formulation so that a more effective target learning model and distribution alignment between local domains and the target domain can be jointly achieved.

For LDA of *m* local domains ${\left\{X_{v}^{s}\right\}}_{v=1}^{m}$ from the source domain *X^s^*, we define the *v*-th (1≤v≤m) local classifier as $f_{v}\left(w_{v},X_{v}^{s}\right)$ corresponding to the *v*-th local domain, where $w_{v}\in {\mathbb{R}}^{d}$ is the *v*-th local classifier model. If we consider kernel learning and assume that there is a feature map $\phi_{v}:\chi \to H_{v}$^1^ that projects the training data from the original feature space into a certain reproducing kernel Hilbert space (RKHS; Gretton et al., 2009) *H_v_*, then the predictor model *w_v_* can be kernelized. We denote the kernel matrix as ${\left(K_{v}\right)}_{i,j}=\phi \left(x_{i}^{v}\right),\;\phi \left(x_{j}^{v}\right)$, where $x_{i}^{v},x_{j}^{v}\in X_{v}^{s}$. We introduce the empirical kernel map as discussed in Pan et al. (2011):


$$\begin{array}{l} \phi_{v}:\chi \to {\mathbb{R}}^{d},for\;linear\;kernel\;mapping\\ \;{x\to K_{v}\left(\cdot ,\;x^{v}\right)|}_{x_{1}^{v},x_{2}^{v},\ldots ,x_{n_{s}}^{v}}=\left(K_{v}\left(x_{1}^{v},\;x^{v}\right),\ldots ,K_{v}(x_{n_{s}}^{v},\;x^{v})\right),\\ \;for\;nonlinear\;kernel\;mapping\; \end{array}$$

We therefore have kernelized data matrices $K_{v}^{s}=\phi_{v}\left(X_{v}^{s}\right)$ for nonlinear projection. For simplicity of expression, we uniformly express the data in linear and nonlinear space as follows:


$${\bar{X}}_{v}^{s}=\left\{ \begin{array}{l} X_{v}^{s},\;\;linear\\ K_{v}^{s}\left(\cdot ,x\right),\;kernel \end{array}\right.$$

In the sequence, we also refer to it as $X_{v}^{s}$ (linear) and $K_{v}^{s}$ (nonlinear) if without special denotation. We further denote by $W=\left[w_{1}\mathrm{;;}\ldots \mathrm{;;}w_{m}\right]$ the concatenated local model matrix. We then endeavor to find *m* local adaptation models parameterized by jointly exploiting correlation information among local domains.

We first formulate our method with classical regularized empirical error (Zhang et al., 2019c), which leads to a classifier *f_v_* based on a set of training data *X_v_*:


(1) $$\min\sum_{v=1}^{m}loss\left(f_{v}\left(w_{v},\;X_{v}\right),y_{v}\right)+\Omega \left(f_{v}\right)$$

where $\Omega \left(f_{v}\right)$ is a regularization term that guarantees good generalization performance and loss(⋅,⋅) is a regression loss function. Although other complex nonlinear models can be used, the linear model has the following characteristics: (1) It is fast and more suitable for practical applications and (2) The local structure of the manifold is approximately linear (Feiping Nie et al., 2010). So, we use the following linear transformation:


(2) $$f_{v}\left(w_{v},\;X_{v}\right)=X_{v}^{T}w_{v}+b_{v}$$

where, $b_{v}\in \mathbb{R}$ is the bias term. The model vectors for all local domains should be highly correlated. So, we further get the following objective function.


(3) $$\begin{array}{l} \underset{\theta_{v},w_{v},b_{v}}{\min}\sum_{v=1}^{m}\left\{\theta_{v}{\;}^{r}{\left\|X_{v}{\;}^{T}w_{v}+b_{v}1_{k_{1}+k_{2}+1}-y_{v}\right\|}_{2}^{2}+\alpha {\left\|w_{v}\right\|}_{2}^{2}\right\}+\beta {\left\|W\right\|}_{*}\\ s.t.\sum_{v=1}^{m}\theta_{v}=1,\theta_{v}\in \left[0,1\right] \end{array}$$

where *α*, *β* is the regularization parameters and the coefficient *θ_v_* is the contribution of each local model. The third term in Eq. (3) is the trace norm of $W\in {\mathbb{R}}^{d\times m}$, which is the convex hull of the rank of *W*, thus enhancing the correlation between different local weight vectors (Yang et al., 2013).

Essentially, it is expected that a bridge needs to be established between different local model vectors. Therefore, we can add a global model vector $\tilde{w}$ and require each local model vector to be aligned with it (Zhang et al., 2019a). Furthermore, to avoid some noise information, we replace the real label vector *y_v_* in Eq. (3) with the pseudo label vector $f_{v}\in {\mathbb{R}}^{k}$. This pseudo-label vector can be obtained by the subsequent optimization. Therefore, the objective function can be represented in the following formulation:


(4) $$\begin{array}{l} \underset{\begin{array}{l} \theta_{v},w_{v},b_{v},\\ b,w \end{array}}{\min}\sum_{v=1}^{n_{+}}\left\{\theta_{v}^{r}{\left\|X_{v}^{T}w_{v}+b_{v}1_{k}-f_{v}\right\|}_{2}^{2}+\alpha {\left\|w_{v}\right\|}_{2}^{2}\right\}+\sum_{v=1}^{n_{+}}{\left\|X_{v}^{T}w_{v}-X_{v}^{T}\tilde{w}\right\|}_{2}^{2}\\ +{\left\|X^{T}\tilde{w}+b1_{n}-f\right\|}_{2}^{2}+{\left\|f-y\right\|}_{2}^{2}+\beta \left({\left\|W\right\|}_{*}+{\left\|\tilde{w}\right\|}_{2}^{2}\right)\\ s.t.\;\;\sum_{v=1}^{n_{+}}\theta_{v}=1,\theta_{v}\in \left[0,1\right] \end{array}$$

where *η* is another regularization parameter. The reason for adding the fifth term is that the predicted results should be consistent with the actual label (Zhang et al., 2020a). We also expect that the local prediction label should be globally consistent, which is obtained by the global weight vector $\tilde{w}$ on each local domain. In other words, the label information should be consistent with the nearby samples.

Given our objectives mentioned above, we propose the following general formulation of LDG:


(5) $$\begin{array}{l} \underset{\begin{array}{l} \theta_{v},w_{v},f,f_{v}\\ b_{v},b,\tilde{w},\lambda_{v} \end{array}}{\min}\sum_{v=1}^{m}\left\{\theta_{v}^{r}{\left\|X_{v}^{T}w_{v}^{\;}+b_{v}^{\;}1_{k}^{\;}-f_{v}^{\;}\right\|}_{2}^{2}+\alpha {\left\|w_{v}^{\;}\right\|}_{2}^{2}\right\}+\sum_{v=1}^{m}{\left\|X_{v}^{T}w_{v}^{\;}-X_{v}^{T}\tilde{w}\right\|}_{2}^{2}\\ +tr\left({\tilde{w}}_{\;}^{T}\left(\sum_{v=1}^{m}\lambda_{v}^{\;}X_{v}^{\;}L_{v}^{\;}X_{v}^{T}\right)\tilde{w}\right)\\ +{\left\|X_{\;}^{T}\tilde{w}+b1_{n}^{\;}-f\right\|}_{2}^{2}+{\left\|f-y\right\|}_{2}^{2}+\beta \left({\left\|W\right\|}_{*}^{\;}+{\left\|\tilde{w}\right\|}_{2}^{2}\right)+\mu \sum_{v=1}^{m}\lambda_{v}^{\;}\log\lambda_{v}^{\;}\\ s.t.\sum_{v=1}^{m}\theta_{v}^{\;}=1,\theta_{v}^{\;}\in \left[0,1\right],\sum_{v=1}^{m}\lambda_{v}^{\;}=1,\lambda_{v}^{\;}\in \left[0,1\right] \end{array}$$

where *λ_v_* is the contribution of different subdomains. In the above equation, the maximum entropy regularization $\lambda_{v}\log\lambda_{v}$ is added to avoid a trivial solution. $L_{v}={\left(E_{v}\right)}^{-1/2}\left(E_{v}-S_{v}\right){\left(E_{v}\right)}^{-1/2}$ is a normalized Laplacian matrix corresponding to the *v*-th local domain (Yan et al., 2006), and *E_v_* is a diagonal matrix with a diagonal element of ${\left(E_{v}\right)}_{i,i}=\sum_{j}^{k}{\left(S_{v}\right)}_{i,j}$. The graph weight matrix *S_v_* of *X_v_* is defined as follows:


$${\left(S_{v}\right)}_{i,j}=\{\begin{array}{ll} \exp\left(-\frac{x_{i}^{v}-x_{j}^{v}{\;}^{2}}{\sigma^{v}}\right), & x_{i}^{v}\in N_{k}\left(x_{j}^{v}\right)\mathrm{or}x_{j}^{v}\in N_{k}\left(x_{i}^{v}\right)\\ 0, & \;\mathrm{otherwise}\; \end{array},$$

where $N_{k}\left(x\right)$ denotes the *k*-NN of *x*.

#### Remark

In our objective formulation, one could adapt the knowledge obtained from multiple local domains to facilitate the target task of interest, which has been empirically demonstrated to be better than learning each local domain task independently in emotion recognition. In other words, it is expected to be beneficial to leverage the common knowledge shared by multiple local domain tasks for AER. However, most of the existing state-of-the-art algorithms uncover some optimal classifier models for the source and/or target domain independently. Moreover, in these state-of-the-art methods, joint multiple local adaptation learning has been largely unaddressed, and little or limited efforts have yet been devoted to the utilization of the correlation information among multiple local domains.

### Optimization

Our objective function is non-smooth, so we propose an alternative algorithm to solve it.

Optimize $b_{v},w_{v},f_{v},f,b$ and $\tilde{w}$ by fixing $\lambda_{v},\theta_{v}$.

By setting the *b_v_* derivative to 0, we have:


(6) $$\begin{array}{l} w_{v}^{T}X_{v}1_{k}+kb_{v}-f_{v}^{T}1_{k}=0\\ \Rightarrow b_{v}=\frac{1}{k}\left(f_{v}^{T}1_{k}-w_{v}^{T}X_{v}1_{k}\right)\\ =\frac{1}{k}\left(1_{k}^{T}f_{v}-1_{v}^{T}X_{v}^{T}w_{v}\right) \end{array}$$

By setting the *b* derivative to 0, we have:


(7) $$\begin{array}{l} {\tilde{w}}^{T}X1_{n}+nb-f^{T}1_{n}=0\\ \Rightarrow b=\frac{1}{n}\left(f^{T}1_{n}-{\tilde{w}}^{T}X1_{n}\right)\\ =\frac{1}{n}\left(1_{n}^{T}f-1_{n}^{T}X^{T}\tilde{w}\right) \end{array}$$

Substituting Eq. (6) and Eq. (7) into Eq. (5), then setting its derivative on *w_v_* to 0, we get the following formula:


(8) $$w_{v}=Q_{v}^{-1}\left(\theta_{v}^{r}X_{v}H_{k}f_{v}+X_{v}X_{v}^{T}\tilde{w}\right)$$

where $H_{k}=I_{k}-\frac{1}{k}1_{k}1_{k}^{T}$, $Q_{v}=\theta_{v}^{r}X_{v}H_{k}X_{v}^{T}+X_{v}X_{v}^{T}+\beta V+\alpha I_{d}$, and $V={\left(W{\left(W\right)}^{T}\right)}^{-1/2}$. By setting the derivative on $\tilde{w}$ to 0, we get:


(9) $$\tilde{w}=A_{v}^{-1}\left(XH_{n}f+\theta_{v}^{r}X_{v}X_{v}^{T}Q_{v}^{-1}X_{v}H_{k}f_{v}\right)$$

where $A_{v}=XH_{n}X^{T}-X_{v}X_{v}^{T}Q_{v}^{-1}X_{v}X_{v}^{T}+X_{v}X_{v}^{T}+\beta I_{d}+\lambda_{v}X_{v}L_{v}X_{v}^{T}$ and $H_{n}=I_{n}-\frac{1}{n}1_{n}1_{n}^{T}$. By setting its derivative for *f_v_* to 0, we get:


(10) $$f_{v}=B_{v}^{-1}\left(\theta_{v}^{r}H_{k}X_{v}^{T}Q_{v}^{-1}X_{v}X_{v}^{T}A_{v}^{-1}XH_{n}f\right)$$

where $\begin{array}{c} B_{v}=\theta_{v}^{r}H_{k}-\theta_{v}^{2r}H_{k}X_{v}^{T}Q_{v}^{-1}X_{v}X_{v}^{T}A^{-1}X_{v}X_{v}^{T}Q_{v}^{-1}X_{v}\\ \;H_{k}-\theta_{v}^{2r}H_{k}X_{v}^{T}Q_{v}^{-1}X_{v}H_{k} \end{array}$. By setting its derivative for *f* to 0, we get:


(11) $$f={\left(\begin{array}{l} I-\theta_{v}^{r}H_{n}X^{T}A_{v}^{-1}X_{v}X_{v}^{T}Q_{v}^{-1}X_{v}H_{k}B_{v}^{-1}\theta_{v}^{r}H_{k}X_{v}^{T}Q_{v}^{-1}\\ X_{v}X_{v}^{T}A_{v}^{-1}XH_{n}-H_{n}X^{T}A_{v}^{-1}XH_{n}+H_{n} \end{array}\right)}^{-1}y$$

Optimize $\theta_{v}{\;}^{r}$ by fixing $b_{v},w_{v},f_{v},\lambda_{v},f,b$ and $\tilde{w}$.

After fixing $b_{v},w_{v},f_{v},\lambda_{v},f,b$ and $\tilde{w}$, the objective function in eq. (5) can be reformulated as


(12) $$\begin{array}{l} \underset{\theta_{v}}{\min}\sum_{v=1}^{m}\left\{\theta_{v}^{r}{\left\|X_{v}^{T}w_{v}+b_{v}1_{k}-f_{v}\right\|}_{2}^{2}\right\}\\ s.t.\sum_{v=1}^{m}\theta_{v}=1,\theta_{v}\in \left[0,1\right], \end{array}$$

By using the Lagrange multiplier *δ*, we convert the above problem into a Lagrange function as follows:


(13) $$M\left(\theta_{v},\delta \right)=\sum_{v=1}^{m}\theta_{v}{\;}^{2}\left({\left\|X_{v}^{T}w_{v}+b_{v}1_{k}-f_{v}\right\|}_{2}^{2}\right)-\delta \left(\sum_{v=1}^{m}\theta_{v}-1\right)$$

By setting its derivative for *θ_i_* to 0, we get:


(14) $$\begin{array}{l} \frac{\partial M}{\partial \theta_{v}}=2\theta_{v}\left({\left\|X_{v}^{T}w_{v}+b_{v}1_{k}-f_{v}\right\|}_{2}^{2}\right)-\delta \\ \theta_{v}=\frac{\delta }{2}\left({\left\|X_{v}^{T}w_{v}+b_{v}1_{k}-f_{v}\right\|}_{2}^{2}\right) \end{array}$$

Since $\sum_{v=1}^{m}\theta_{v}=1$, we obtain:


(15) $$\theta_{v}=\frac{\theta_{v}}{\sum_{v=1}^{m}\theta_{v}}=\frac{1/\left({\left\|X_{v}^{T}w_{v}+b_{v}1_{k}-f_{v}\right\|}_{2}^{2}\right)}{\sum_{v=1}^{m}1/\left({\left\|X_{v}^{T}w_{v}+b_{v}1_{k}-f_{v}\right\|}_{2}^{2}\right)}$$

Optimize *λ_v_* by fixing $b_{v},w_{v},f_{v},\theta_{v},f,b$ and $\tilde{w}$.

When fixing $b_{v},w_{v},f_{v},\theta_{v},f,b$ and $\tilde{w}$, the objective function in Eq. (5) is equivalent to:


(16) $$\begin{array}{l} \underset{\lambda_{v}}{\min}tr\left({\tilde{w}}^{T}\left(\sum_{v=1}^{m}\lambda_{v}X_{v}L_{v}X_{v}^{T}\right)\tilde{w}\right)+\mu \sum_{v=1}^{m}\lambda_{v}\log\lambda_{v}\\ s.t.\sum_{v=1}^{m}\lambda_{v}=1,\lambda_{v}\in \left[0,1\right] \end{array}$$

By using the Lagrange multiplier *φ*, we convert the above problem into a Lagrange function as follows:


(17) $$\begin{array}{l} L\left(\lambda_{v},\varphi \right)=tr\left({\tilde{w}}^{T}\left(\sum_{v=1}^{m}\lambda_{v}X_{v}L_{v}X_{v}^{T}\right)\tilde{w}\right)\\ \;+\mu \sum_{v=1}^{m}\lambda_{v}\log\lambda_{v}-\varphi \left(\sum_{v=1}^{m}\lambda_{v}-1\right) \end{array}$$

By setting its derivative for *λ_v_* to 0, we have:


$$tr\left({\tilde{w}}^{T}X_{v}L_{v}X_{v}^{T}\tilde{w}\right)+\mu \log\lambda_{v}+\mu -\varphi =0$$

We thus obtain:


(18) $$\lambda_{v}=\frac{\lambda_{v}}{\sum_{v=1}^{m}\lambda_{v}}=\frac{\exp\left(\left(-tr\left({\tilde{w}}^{T}X_{v}L_{v}X_{v}^{T}\tilde{w}\right)-\mu \right)/\mu \right)}{\sum_{v=1}^{M}\exp\left(\left(-tr\left({\tilde{w}}^{T}X_{v}L_{v}X_{v}^{T}\tilde{w}\right)-\mu \right)/\mu \right)}$$

### Overall algorithm and convergence analysis

According to the above objective function optimization process, we summarize the following algorithm for LDG.

Below, we will demonstrate that the alternating optimization procedure converges to the optimal solution of ${\left\{w_{v}\right\}}_{v=1}^{m}$ corresponding to the optimization problem (5) according to Lemma 1.

**Lemma 1.** For any invertible matrices *M* and $\tilde{V}$, the following inequality holds (Nie et al., 2010):

(19) $$\frac{1}{2}tr\left(M{\tilde{V}}^{-\frac{1}{2}}\right)-tr\left(M^{\frac{1}{2}}\right)\geq \frac{1}{2}tr\left(\tilde{V}{\tilde{V}}^{-\frac{1}{2}}\right)-tr\left({\tilde{V}}^{\frac{1}{2}}\right)$$

Next, we verify that the proposed iterative approach in Algorithm 1 can converge to the optimal solutions by the following theorem:

**Theorem 1.** Algorithm 1 will monotonically decrease the objective of the problem in Eq. (5) in each iteration and will converge to the optimum of the problem.

**Proof**. For ease of representation, we denote the updated $b_{v},w_{v},f_{v},\theta_{v},\lambda_{v}$, *b*, and $\tilde{w}$ in each iteration as $b_{v}^{l},w_{v}^{l},f_{v}^{l},\theta_{v}^{l},\lambda_{v}^{l},f^{l},b^{l}$ and ${\tilde{w}}^{l}$, respectively. The inner loop to update in Step 2 of Algorithm 1 corresponds to the optimization of the following problem.

(20) $$\begin{array}{l} \underset{\begin{array}{l} \theta_{v},w_{v},f,f_{v}\\ b_{v},b,\tilde{w},\lambda_{v} \end{array}}{\min}\sum_{v=1}^{n_{+}}\left\{\theta_{v}^{r}{\left\|X_{v}^{T}w_{v}^{\;}+b_{v}^{\;}1_{k}^{\;}-f_{v}^{\;}\right\|}_{2}^{2}+\alpha {\left\|w_{v}^{\;}\right\|}_{2}^{2}\right\}\\ +\sum_{v=1}^{n_{+}}{\left\|X_{v}^{T}w_{v}^{\;}-X_{v}^{T}\tilde{w}\right\|}_{2}^{2}+tr\left({\tilde{w}}_{\;}^{T}\left(\sum_{v=1}^{n_{+}}\lambda_{v}^{\;}X_{v}^{\;}L_{v}^{\;}X_{v}^{T}\right)\tilde{w}\right)\\ +{\left\|X_{\;}^{T}\tilde{w}+b1_{n}^{\;}-f\right\|}_{2}^{2}+{\left\|f-y\right\|}_{2}^{2}+\beta \left({\left\|W\right\|}_{*}^{\;}+{\left\|\tilde{w}\right\|}_{2}^{2}\right)\\ +\mu \sum_{v=1}^{n_{+}}\lambda_{v}^{\;}\log\lambda_{v}^{\;} \end{array}$$

**Table:** 

**Algorithm 1: Local domain generalization and adaptation**
**Input:** Domain training dataset $\left\{\left(X_{v},y_{v}\right),v=1,\ldots ,m\right\}$; the number of nearest neighbors *k*_1_, *k*_2_, and parameters *α*, *β*, and *μ*. **Initialization:** Set *t* = 0, and initialize $w_{v}^{0}$, $f_{v}^{0}$, $b_{v}^{0}$, ${\tilde{w}}^{0}$, $\theta_{v}{\;}^{0}$, $\lambda_{v}{\;}^{0}$, $b^{0}$, $f^{0}$ randomly, and set $V_{v}^{0}$ as identity matrix. 1: Construct the *k*-nearest neighbor graph and calculate ${\left\{L_{v}\right\}}_{v=1}^{M}$; 2:Compute *H_k_* according to $H_{k}=I_{k}-\frac{1}{k}1_{k}1_{k}^{T}$; 3:Compute *H_n_* according to $H_{n}=I_{n}-\frac{1}{n}1_{n}1_{n}^{T}$; 4: For each *v* in {1,…,m} { 4.1: Let *t* = 0; 4.2: repeat { 4.2.1:Compute $\lambda_{v}^{t}$ according to Eq. (18); 4.2.2:Obtain $\theta_{v}^{t}$ by Eq. (15); 4.2.3: Compute $Q_{v}^{t}$ as $Q_{v}^{t}={\left(\theta_{v}^{t}\right)}^{r}X_{v}H_{k}X_{v}^{T}+X_{v}X_{v}^{T}+\beta V^{t}+\alpha I_{d}$; 4.2.4: Update $A_{v}^{t}$ as $A_{v}^{t}=XH_{n}X^{T}-X_{v}X_{v}^{T}{\left(A_{v}^{t}\right)}^{-1}X_{v}X_{v}^{T}+X_{v}X_{v}^{T}+\beta I_{d}+\lambda_{v}^{t}X_{v}L_{v}X_{v}^{T}$; 4.2.5: Update $B_{v}^{t}$ as $\begin{array}{c} B_{v}^{t}={\left(\theta_{v}^{t}\right)}^{r}H_{k}-{\left(\theta_{v}^{t}\right)}^{2r}H_{k}X_{v}^{T}{\left(Q_{v}^{t}\right)}^{-1}X_{v}X_{v}^{T}{\left(A_{v}^{t}\right)}^{-1}X_{v}X_{v}^{T}{\left(Q_{v}^{t}\right)}^{-1}X_{v}H_{k}\\ -{\left(\theta_{v}^{t}\right)}^{2r}H_{k}X_{v}^{T}{\left(Q_{v}^{t}\right)}^{-1}X_{v}H_{k} \end{array}$; 4.2.6: Compute $f^{0}$ according to Eq. (11); 4.2.7: Compute $f_{v}^{t}$ according to Eq. (10); 4.2.8: Update ${\tilde{w}}^{t}$ as ${\tilde{w}}^{t}=A_{v}^{-1}\left(XH_{n}f^{t}+{\left(\theta_{v}^{t}\right)}^{r}X_{v}X_{v}^{T}{\left(Q_{v}^{t}\right)}^{-1}X_{v}H_{k}f_{v}^{t}\right)$; 4.2.9: Update $w_{v}^{t}$ as $w_{v}^{t}={\left(Q_{v}^{t}\right)}^{-1}\left({\left(\theta_{v}^{t}\right)}^{r}X_{v}H_{k}f_{v}^{t}+X_{v}X_{v}^{T}{\tilde{w}}^{t}\right)$; 4.2.10: Update $b_{v}^{t}$ as $b_{v}^{t}=\frac{1}{k}\left(1_{k}^{T}f_{v}^{t}-1_{v}^{T}X_{v}^{T}w_{v}^{t}\right)$; 4.2.11: Update $b_{v}^{t}$ as $b^{t}=\frac{1}{n}\left(1_{n}^{T}f^{t}-1_{n}^{T}X^{T}{\tilde{w}}^{t}\right)$; 4.2.12: Update $V_{v}^{t}$; 4.2.13: Set t=t+1; } until $\left|\max\Omega_{t}-\min\Omega_{t}\right|/\max\Omega_{t}<10^{-4}$; 4.3 Next v; } **Output:** Converged $\lambda_{v}$, $\theta_{v}$, $w_{v}$, $b_{v}$, $b_{v}$, $\tilde{w}$, f, $f_{v}$.

According to the definitions of the matrix *V*, we obtain:


(21) $$\begin{array}{l} \underset{\begin{array}{l} \theta_{v},w_{v},f,f_{v}\\ b_{v},b,\tilde{w},\lambda_{v} \end{array}}{\min}\sum_{v=1}^{M}\left\{{\left(\theta_{v}{\;}^{l+1}\right)}^{r}{\left\|X_{v}^{T}w_{v}^{l+1}+b_{v}^{l+1}1_{k}^{\;}-f_{v}^{l+1}\right\|}_{2}^{2}+\alpha {\left\|w_{v}^{l+1}\right\|}_{2}^{2}\right\}\\ +\sum_{v=1}^{M}{\left\|X_{v}^{T}w_{v}^{l+1}-X_{v}^{T}{\tilde{w}}^{l+1}\right\|}_{2}^{2}+tr\left({\left({\tilde{w}}^{l+1}\right)}_{\;}^{T}\left(\sum_{v=1}^{M}\lambda_{v}^{l+1}X_{v}^{\;}L_{v}^{\;}X_{v}^{T}\right){\tilde{w}}^{l+1}\right)\\ +{\left\|X_{\;}^{T}{\tilde{w}}^{l+1}+b^{l+1}1_{n}^{\;}-f^{l+1}\right\|}_{2}^{2}+{\left\|f^{l+1}-y\right\|}_{2}^{2}+\beta \left({\left\|W^{l+1}\right\|}_{*}^{\;}+{\left\|{\tilde{w}}^{l+1}\right\|}_{2}^{2}\right)\\ +\mu \sum_{v=1}^{M}\lambda_{v}^{{\;}^{l+1}}\log\lambda_{v}^{{\;}^{l+1}}\\ \leq \underset{\begin{array}{l} \theta_{v},w_{v},f,f_{v}\\ b_{v},b,\tilde{w},\lambda_{v} \end{array}}{\min}\sum_{v=1}^{M}\left\{{\left(\theta_{v}{\;}^{l}\right)}^{r}{\left\|X_{v}^{T}w_{v}^{l}+b_{v}^{l}1_{k}^{\;}-f_{v}^{l}\right\|}_{2}^{2}+\alpha {\left\|w_{v}^{l}\right\|}_{2}^{2}\right\}\\ +\sum_{v=1}^{M}{\left\|X_{v}^{T}w_{v}^{l}-X_{v}^{T}{\tilde{w}}^{l}\right\|}_{2}^{2}+tr\left({\left({\tilde{w}}^{l}\right)}_{\;}^{T}\left(\sum_{v=1}^{M}\lambda_{v}^{l}X_{v}^{\;}L_{v}^{\;}X_{v}^{T}\right){\tilde{w}}^{l}\right)\\ +{\left\|X_{\;}^{T}{\tilde{w}}^{l}+b^{l}1_{n}^{\;}-f^{l}\right\|}_{2}^{2}+{\left\|f^{l}-y\right\|}_{2}^{2}+\beta \left({\left\|W^{l}\right\|}_{*}^{\;}+{\left\|{\tilde{w}}^{l}\right\|}_{2}^{2}\right)\\ +\mu \sum_{v=1}^{M}\lambda_{v}^{{\;}^{l}}\log\lambda_{v}^{{\;}^{l}} \end{array}$$

Eq. (21) is equivalent to the following form:


(22) $$\begin{array}{l} \underset{\begin{array}{l} \theta_{v},w_{v},f,f_{v}\\ b_{v},b,\tilde{w},\lambda_{v} \end{array}}{\min}\sum_{v=1}^{M}\left\{{\left(\theta_{v}{\;}^{l+1}\right)}^{r}{\left\|X_{v}^{T}w_{v}^{l+1}+b_{v}^{l+1}1_{k}^{\;}-f_{v}^{l+1}\right\|}_{2}^{2}+\alpha {\left\|w_{v}^{l+1}\right\|}_{2}^{2}\right\}\\ +\sum_{v=1}^{M}{\left\|X_{v}^{T}w_{v}^{l+1}-X_{v}^{T}{\tilde{w}}^{l+1}\right\|}_{2}^{2}+tr\left({\left({\tilde{w}}^{l+1}\right)}_{\;}^{T}\left(\sum_{v=1}^{M}\lambda_{v}^{l+1}X_{v}^{\;}L_{v}^{\;}X_{v}^{T}\right){\tilde{w}}^{l+1}\right)\\ +{\left\|X_{\;}^{T}{\tilde{w}}^{l+1}+b^{l+1}1_{n}^{\;}-f^{l+1}\right\|}_{2}^{2}+{\left\|f^{l+1}-y\right\|}_{2}^{2}+\beta {\left\|{\tilde{w}}^{l+1}\right\|}_{2}^{2}\\ +\mu \sum_{v=1}^{M}\lambda_{v}^{{\;}^{l+1}}\log\lambda_{v}^{{\;}^{l+1}}+\beta tr\left(W^{l+1}{\left(W^{l+1}\right)}^{T}V^{l+1}\right)-\frac{\beta }{2}\mathrm{tr}\left({\left(W^{l+1}{\left(W^{l+1}\right)}^{T}\right)}^{\frac{1}{2}}\right)\\ +\frac{\beta }{2}\mathrm{tr}\left({\left(W^{l+1}{\left(W^{l+1}\right)}^{T}\right)}^{\frac{1}{2}}\right)\\ \leq \underset{\begin{array}{l} \theta_{v},w_{v},f,f_{v}\\ b_{v},b,\tilde{w},\lambda_{v} \end{array}}{\min}\sum_{v=1}^{M}\left\{{\left(\theta_{v}{\;}^{l}\right)}^{r}{\left\|X_{v}^{T}w_{v}^{l}+b_{v}^{l}1_{k}^{\;}-f_{v}^{l}\right\|}_{2}^{2}+\alpha {\left\|w_{v}^{l}\right\|}_{2}^{2}\right\}\\ +\sum_{v=1}^{M}{\left\|X_{v}^{T}w_{v}^{l}-X_{v}^{T}{\tilde{w}}^{l}\right\|}_{2}^{2}+tr\left({\left({\tilde{w}}^{l}\right)}_{\;}^{T}\left(\sum_{v=1}^{M}\lambda_{v}^{l}X_{v}^{\;}L_{v}^{\;}X_{v}^{T}\right){\tilde{w}}^{l}\right)\\ +{\left\|X_{\;}^{T}{\tilde{w}}^{l}+b^{l}1_{n}^{\;}-f^{l}\right\|}_{2}^{2}+{\left\|f^{l}-y\right\|}_{2}^{2}+\beta {\left\|{\tilde{w}}^{l}\right\|}_{2}^{2}\\ +\mu \sum_{v=1}^{M}\lambda_{v}^{{\;}^{l}}\log\lambda_{v}^{{\;}^{l}}+\beta tr\left(W^{l}{\left(W^{l}\right)}^{T}V^{l}\right)-\frac{\beta }{2}\mathrm{tr}\left({\left(W^{l}{\left(W^{l}\right)}^{T}\right)}^{\frac{1}{2}}\right)\\ +\frac{\beta }{2}\mathrm{tr}\left({\left(W^{l}{\left(W^{l}\right)}^{T}\right)}^{\frac{1}{2}}\right) \end{array}$$

Since $V^{l}=\frac{1}{2}{\left(W^{l}{\left(W^{l}\right)}^{T}\right)}^{-\frac{1}{2}}$ and according to Lemma 1, we obtain:


(23) $$\begin{array}{l} \beta tr\left(W^{l+1}{\left(W^{l+1}\right)}^{T}V^{l+1}\right)-\beta tr\left({\left(W^{l+1}{\left(W^{l+1}\right)}^{T}\right)}^{\frac{1}{2}}\right)\\ \geq \beta tr\left(W^{l}{\left(W^{l}\right)}^{T}V^{l}\right)-\beta tr\left({\left(W^{l}{\left(W^{l}\right)}^{T}\right)}^{\frac{1}{2}}\right) \end{array}$$

Subtracting (23) from (22), we have:


(24) $$\begin{array}{l} \underset{\begin{array}{l} \theta_{v},w_{v},f,f_{v}\\ b_{v},b,\tilde{w},\lambda_{v} \end{array}}{\min}\sum_{v=1}^{M}\left\{{\left(\theta_{v}{\;}^{l+1}\right)}^{r}{\left\|X_{v}^{T}w_{v}^{l+1}+b_{v}^{l+1}1_{k}^{\;}-f_{v}^{l+1}\right\|}_{2}^{2}+\alpha {\left\|w_{v}^{l+1}\right\|}_{2}^{2}\right\}\\ +\sum_{v=1}^{M}{\left\|X_{v}^{T}w_{v}^{l+1}-X_{v}^{T}{\tilde{w}}^{l+1}\right\|}_{2}^{2}\\ +tr\left({\left({\tilde{w}}^{l+1}\right)}_{\;}^{T}\left(\sum_{v=1}^{M}\lambda_{v}^{l+1}X_{v}^{\;}L_{v}^{\;}X_{v}^{T}\right){\tilde{w}}^{l+1}\right)\\ +{\left\|X_{\;}^{T}{\tilde{w}}^{l+1}+b^{l+1}1_{n}^{\;}-f^{l+1}\right\|}_{2}^{2}+{\left\|f^{l+1}-y\right\|}_{2}^{2}+\beta {\left\|{\tilde{w}}^{l+1}\right\|}_{2}^{2}\\ +\mu \sum_{v=1}^{M}\lambda_{v}^{{\;}^{l+1}}\log\lambda_{v}^{{\;}^{l+1}}+\frac{\beta }{2}tr\left({\left(W^{l+1}{\left(W^{l+1}\right)}^{T}\right)}^{\frac{1}{2}}\right)\\ \leq \underset{\begin{array}{l} \theta_{v},w_{v},f,f_{v}\\ b_{v},b,\tilde{w},\lambda_{v} \end{array}}{\min}\sum_{v=1}^{M}\left\{{\left(\theta_{v}{\;}^{l}\right)}^{r}{\left\|X_{v}^{T}w_{v}^{l}+b_{v}^{l}1_{k}^{\;}-f_{v}^{l}\right\|}_{2}^{2}+\alpha {\left\|w_{v}^{l}\right\|}_{2}^{2}\right\}\\ +\sum_{v=1}^{M}{\left\|X_{v}^{T}w_{v}^{l}-X_{v}^{T}{\tilde{w}}^{l}\right\|}_{2}^{2}+tr\left({\left({\tilde{w}}^{l}\right)}_{\;}^{T}\left(\sum_{v=1}^{M}\lambda_{v}^{l}X_{v}^{\;}L_{v}^{\;}X_{v}^{T}\right){\tilde{w}}^{l}\right)\\ +{\left\|X_{\;}^{T}{\tilde{w}}^{l}+b^{l}1_{n}^{\;}-f^{l}\right\|}_{2}^{2}+{\left\|f^{l}-y\right\|}_{2}^{2}+\beta {\left\|{\tilde{w}}^{l}\right\|}_{2}^{2}+\mu \sum_{v=1}^{M}\lambda_{v}^{{\;}^{l}}\log\lambda_{v}^{{\;}^{l}}\\ +\frac{\beta }{2}tr\left({\left(W^{l}{\left(W^{l}\right)}^{T}\right)}^{\frac{1}{2}}\right) \end{array}$$

The above formula is equivalent to:


(25) $$\begin{array}{l} \underset{\begin{array}{l} \theta_{v},w_{v},f,f_{v}\\ b_{v},b,\tilde{w},\lambda_{v} \end{array}}{\min}\sum_{v=1}^{M}\left\{{\left(\theta_{v}{\;}^{l+1}\right)}^{r}{\left\|X_{v}^{T}w_{v}^{l+1}+b_{v}^{l+1}1_{k}^{\;}-f_{v}^{l+1}\right\|}_{2}^{2}+\alpha {\left\|w_{v}^{l+1}\right\|}_{2}^{2}\right\}\\ +\sum_{v=1}^{M}{\left\|X_{v}^{T}w_{v}^{l+1}-X_{v}^{T}{\tilde{w}}^{l+1}\right\|}_{2}^{2}\\ +tr\left({\left({\tilde{w}}^{l+1}\right)}_{\;}^{T}\left(\sum_{v=1}^{M}\lambda_{v}^{l+1}X_{v}^{\;}L_{v}^{\;}X_{v}^{T}\right){\tilde{w}}^{l+1}\right)+{\left\|X_{\;}^{T}{\tilde{w}}^{l+1}+b^{l+1}1_{n}^{\;}-f^{l+1}\right\|}_{2}^{2}\\ +{\left\|f^{l+1}-y\right\|}_{2}^{2}\\ +\beta {\left\|{\tilde{w}}^{l+1}\right\|}_{2}^{2}+\mu \sum_{v=1}^{M}\lambda_{v}^{{\;}^{l+1}}\log\lambda_{v}^{{\;}^{l+1}}+\beta {\left\|W^{l+1}\right\|}_{*}\\ \leq \underset{\begin{array}{l} \theta_{v},w_{v},f,f_{v}\\ b_{v},b,\tilde{w},\lambda_{v} \end{array}}{\min}\sum_{v=1}^{M}\left\{{\left(\theta_{v}{\;}^{l}\right)}^{r}{\left\|X_{v}^{T}w_{v}^{l}+b_{v}^{l}1_{k}^{\;}-f_{v}^{l}\right\|}_{2}^{2}+\alpha {\left\|w_{v}^{l}\right\|}_{2}^{2}\right\}\\ +\sum_{v=1}^{M}{\left\|X_{v}^{T}w_{v}^{l}-X_{v}^{T}{\tilde{w}}^{l}\right\|}_{2}^{2}+tr\left({\left({\tilde{w}}^{l}\right)}_{\;}^{T}\left(\sum_{v=1}^{M}\lambda_{v}^{l}X_{v}^{\;}L_{v}^{\;}X_{v}^{T}\right){\tilde{w}}^{l}\right)\\ +{\left\|X_{\;}^{T}{\tilde{w}}^{l}+b^{l}1_{n}^{\;}-f^{l}\right\|}_{2}^{2}+{\left\|f^{l}-y\right\|}_{2}^{2}+\beta {\left\|{\tilde{w}}^{l}\right\|}_{2}^{2}+\mu \sum_{v=1}^{M}\lambda_{v}^{{\;}^{l}}\log\lambda_{v}^{{\;}^{l}}+\beta {\left\|W^{l}\right\|}_{*} \end{array}$$

Therefore, we have proved the theorem. Because of the updating rule in Algorithm 1, the objective function shown in (5) monotonically decreases, and it is easy to see that the algorithm converges.

### Target inference

After training the LDG, we get *m* local classifiers. In the following sections, we will separately propose ways to effectively use these learned classifiers in two cases.

1. LDG: The first is a domain generalization scenario where the target domain samples are not available during training. The other is the domain adaptation scenario with a specific target domain in which we have unlabeled data in it during the training process. In the domain generalization scenario, under the premise that we have no prior information about the target domain, we can only fuse the *m* local classifiers to achieve the prediction of the test sample by assigning different weights. Given a target sample *x*, the predictive label *y* can be obtained by the following formula.
  (26) $$y=\sum_{v=1}^{m}\theta_{v}^{2}f_{v}\left(w_{v},X_{v}\right)=\sum_{v=1}^{m}\theta_{v}^{2}\left(x^{T}w_{v}+b_{v}\right)$$
LDA: When there is unlabeled data in the target domain, we can assign different weights to each local classifier by measuring the similarity between the target domain and each locality in the source domain to achieve a better prediction effect. In other words, when a certain local domain is closer to the target domain, we should assign a higher weight to the classifier trained on this subdomain, and vice versa.

Given a set of target domain samples $X=\left\{x_{1},x_{2},\ldots ,x_{K}\right\}$, where *K* is the number of samples in the target domain. By measuring the distance between the training sample and the target domain by the Maximum Mean Discrepancy (MMD), we get the following formula:


(27) $$\Psi^{v}=Dist\left(X_{v},X\right)=\frac{1}{k}\sum_{i=1}^{k}\phi \left(x_{i}^{v}\right)-\frac{1}{K}\sum_{j=1}^{K}\phi {\left(x_{j}\right)}_{H_{k}}$$

where *X_v_*, *X* are the *v*-th local source domain and target domain datasets respectively, and $Dist\left(X_{v},X\right)$ represents the distribution distance of *X_v_* and *X*, and *H_K_* denotes a regenerative kernel Hilbert space. $\phi \left(\cdot \right)$ is a Gaussian kernel nonlinear feature mapping function. Using MMD we can get the weight of each local classifier by:


(28) $$\zeta_{v}=\frac{\exp\left(-\Psi^{v}\right)}{\sum_{v=1}^{m}\exp\left(-\Psi^{v}\right)},v=1,2,\cdots ,m$$

Then we can predict the test sample *x_j_* by the following formula:


(29) $$y_{j}=\sum_{v}\zeta_{v}\left(x_{j}^{T}w_{v}+b_{v}\right)$$

## Experimental results

In this section, we will conduct comprehensive experiments to validate the effectiveness of our method compared with several state-of-the-art ones.

### Benchmark datasets

Two widely used benchmark databases, i.e., SEED (Zheng and Lu, 2015) and DEAP (Koelstra et al., 2012), are adopted for systematic experiments of EEG-based emotion recognition (Dan et al., 2021; Tao et al., 2022). More detailed descriptions of these two benchmarks can be found in Lan et al. (2018). As reported by references (Zhong P. et al., 2020; Zhong Q. et al., 2020) and (Lan et al., 2018), some obvious differences between these two benchmarks are that they may be sampled from multiple different sources such as different sessions, subjects, experimental schemes, EEG devices, and emotional stimuli, etc. Following the same practice in literature (Shi et al., 2013; Zheng et al., 2015; Chai et al., 2016, 2017; Zheng and Lu, 2016; Lan et al., 2018; Zhong P. et al., 2020; Zhong Q. et al., 2020; Tao and Dan, 2021; Tao et al., 2022) for domain adaptation emotion recognition, differential entropy (DE; Lan et al., 2018; Zhong P. et al., 2020; Zhong Q. et al., 2020) is adopted as the data feature in our experimental settings.

### Baselines and protocol

#### Baselines

As a DG method, we compare our method with several representative domain generalization/adaptation methods, which can be summarized into the following two groups (here we only report the better models):

1. Shallow learning methods: Undo-Bias (Khosla et al., 2012), UML (Fang et al., 2013), DICA (Muandet et al., 2013), LRE-SVM (Xu et al., 2014), and SCA (Ghifary et al., 2017);
2. Deep learning methods: Deep subdomain adaptation network (DSAN; Zhu et al., 2020), Deep domain generalization with structured low-rank constraint (DDG) (Ding et al., 2018a,b,c), deep domain confusion (DDC) (Tzeng et al., 2014), domain adversarial neural networks (DANNs) (Ganin et al., 2016), contrastive adaptation network (CAN) (Kang et al., 2022), and deep CORAL (Sun and Saenko, 2016).

#### Training protocol

For all datasets, we only exploit the source samples for training. We use support vector machine (SVM) as the base classifier for DICA and SCA. The tunable hyper-parameters are selected according to labels from the source domain. We adopt the Gaussian kernel with a kernel bandwidth *σ* computed by the median heuristic as the kernel function for the kernel-based methods. For a fair comparison, all deep learning baselines use the same architecture (ResNet101; He et al., 2016). That is, for deep domain generalization on the EEG dataset, we employed the Resnet101 architecture to extract the training features. We fine-tune all convolutional and pooling layers from pre-trained models and train the classifier layer via back-propagation. For multi-class classification of emotion recognition, we employ the “One vs. Rest” strategy to train our method (Zhang et al., 2020b).

#### Parameter setting

There are several vital parameters such as *μ*, *α*, and *β* that need to be determined beforehand in our objective (5) since they are employed to balance the importance of structure characterization and regularizers. Considering that parameter determination is a yet unaddressed open issue in the field of machine learning, we determine them by grid search in a heuristic way (Nie et al., 2010; Long et al., 2014b; Tao et al., 2022). Concretely, these regularization parameters are tuned from $\left\{10^{-4},10^{-3},\ldots ,10^{3},10^{4}\right\}$. Since no target labels are available for DG, it is impossible to conduct a standard cross-validation. Hence, we perform *p*-fold cross-validation on the labeled source subdomains, namely, calculating the averaged accuracy on each subdomain fold while exploiting the other *p* − 1 subdomain folds for training. Moreover, for constructing the nearest neighbor graph in LDG, we search the optimal neighbor number *k* (including *k*_1_ and *k*_1_) in the grid {3,5,7,9,11,13}, and then report the top-one recognition accuracy from the best parameter configuration. For the kernel learning scenarios, the Gaussian kernel, i.e., $K_{i,j}=\exp\left(-\sigma {\left\|x_{i}-x_{j}\right\|}^{2}\right)$, is used as the default kernel function, where *σ* is set to 1/*d* (*d* is the feature dimension).

### Inter-subject domain generalization

Note that different subjects even from the same dataset still have different EEG feature distributions due to their characteristics. We therefore conduct the so-called leave-one-out cross-validation strategy conducted also in Lan et al. (2018) and Tao et al. (2022) to evaluate the emotion recognition performance. That is, one subject remains to be the target domain, and others from the dataset are constructed as the source domain. In this scenario, we follow the same setting as (Lan et al., 2018; Tao and Dan, 2021; Tao et al., 2022) to evaluate our method compared with other state-of-the-art methods on SEED and DEAP, respectively.

Each subject from DEAP includes 180 samples belonging to three categories, i.e., 60 samples per class. Each subject from SEED contributes 2,775 samples, i.e., 925 samples per class and per session. Following the same strategy adopted by Chai et al. (2016), Zheng and Lu (2016), and Chai et al. (2017), we randomly sampled 1/10 of the training data (3,885 samples contributed by 14 subjects) from SEED in each experiment due to the large number of training samples. To cover the whole training dataset, we randomly extracted 10 training sets from SEED and thus conducted each experimental procedure 10 times. The final result was averaged over these 10 runs. We compared the performance of our LDG with several state-of-the-art DG approaches. The mean recognition accuracies of LDG compared with the baselines on two benchmark datasets are recorded in Table 2.

**Table 2:** Inter-subject recognition accuracy (mean % and STD %).

Method		DEAP		SEED							
				Session I		Session II		Session III		Average	
		Mean	STD	Mean	STD	Mean	STD	Mean	STD	Mean	STD
Shallow methods	Undo-Bias	60.36	3.41	69.41	5.44	65.79	2.24	72.64	5.10	69.28	4.26
	UML	62.18	4.09	72.57	6.27	67.58	1.75	71.17	3.68	70.44	3.90
	DICA	65.33	6.22	73.12	6.86	65.06	6.28	73.38	7.19	70.52	6.78
	LRE-SVM	68.20	2.12	77.50	3.29	70.11	5.44	77.45	4.53	75.02	4.42
	SCA	66.05	4.26	75.23	5.17	69.14	6.20	74.23	6.07	72.87	5.81
	LDG	71.51	3.14	78.92	5.65	70.88	5.72	78.93	5.38	76.24	5.58
Deep methods	DDG	77.68	3.33	84.92	6.42	74.29	7.45	82.33	8.11	80.51	7.33
	DDC	74.87	6.28	79.43	7.13	72.16	6.11	80.07	7.66	77.22	6.97
	DANN	75.34	7.11	82.51	6.49	73.77	7.59	83.62	6.51	79.97	6.86
	DSAN	78.44	4.15	84.50	6.18	74.58	6.33	84.10	6.12	81.06	6.21
	CORAL	74.08	3.58	80.42	4.20	71.54	5.49	81.00	5.00	77.65	4.90
	CAN	78.43	6.10	85.77	7.31	74.12	7.50	85.39	7.40	81.76	7.40
	LDG + Resnet101	77.62	5.37	85.42	5.72	74.68	5.19	86.05	6.82	82.05	5.91
Upp Bnd of Chn Lvl (UBCL)		38.85		34.58		34.65		34.60		34.61	

Bold denotes the best recognition rates.

As is known, when the size of training data increases to infinity, the theoretical performance (about 33.33%) of the random prediction can be approximately approached by the real chance level (Lan et al., 2018). When there are finite samples, we obtain the empirical chance level by repeating the trials with the samples in question equipped with randomized class labels (Lan et al., 2018). For comparison, we also provided the upper bound of chance levels (UBCL) with a 95% confidence interval in this table.

### Comparison with shallow methods

As observed from Table 2, the mean performance of all methods on two datasets has significantly exceeded UBCL at a 5% significance level. This indicates the imperative importance of inter-subject domain generalization due to the intrinsic existence of distribution divergence among different subjects. Compared with other shallow learning methods, our method LDG undoubtedly obtains the best mean performance (75.06% ± 4.97) in all cases, which is followed by LRE-SVM (73.32% ± 3.85). This may be attributed to the subdomain learning technologies in LDG and LRE-SVM. Our method LDG unsurprisingly achieved more performance gains than LRE-SVM on both DEAP and SEED. The multi-source generalization method SCA and DICA are found to be more effective than Undo-bias and UML. The experimental results in Table 2 show that while the relative improvement over vanilla DA/DG methods is significant (*t*-test, value of *p* > 0.05), the absolute accuracy is still rather low, which suggests that there still exists adverse impact incurred by substantial distribution discrepancies between different subjects.

An interesting result that can be observed from Table 2 is that all methods demonstrate better performance on SEED than on DEAP. The same observation has also been reported in Lan et al. (2018) and Tao and Dan (2021). A possible explanation for this result might be that there exist large discrepancies among different subjects, and the samples are distributed more “orderly” in their original feature space on SEED than that on DEAP (Mansour et al., 2009), thus leading to better alignment on SEED in some kernel space. That is, larger discrepancies among different subjects from DEAP could degrade the recognition accuracy to some extent (Mansour et al., 2009; Lan et al., 2018).

### Comparison with deep methods

Following the same settings in Donahue et al. (2014) and Zhou et al. (2022), our method LDG also can be easily trained with the deeply extracted features via the classical deep models such as VGG (Simonyan and Zisserman, 2014) and ResNet (He et al., 2016). Specifically, one can fine-tune some pre-trained deep models (e.g., Resnet101; He et al., 2016) through the source domain, and then extract the deep features from EEG signals. Finally, the recognition model can be learned using these deep representations.

In this part, we will particularly evaluate our method LDG with deeply extracted features by comparing it with several recently proposed deep adaptation models. We additionally denote our method with deep features as LDG + ResNet101. As for other deep benchmarks, their opened source codes are directly borrowed to fine-tune the pre-trained models adopted in their works, respectively. Different from these deep adaptation models, which typically pursue gaining certain domain-invariant representations, our proposed method explores optimizing a domain-invariant recognition model with strong generalization ability from the single source domain to the unseen target. We expect our method leveraging the deeply extracted features can further upgrade the recognition performance with the proposed subdomain generalization strategy.

As shown in Table 2, all of the deep methods obviously outperform the shallow ones. This indicates the advantage of deep features due to their more discriminative representations. As expected, LDG + ResNet101 also obtains the best or comparable recognition performance compared with other deep adaptation methods, followed by CAN and DSAN. This may be partly attributed to the classification-level modeling in our LDG, where most of the local discriminative structures have been preserved by the guidance of subdomain construction. In some scenarios, shown in Table 2, LDG + ResNet101 even achieves the top-one accuracy, which verifies that the proposed LDG can become an effective surrogate to the deep adaptation model by exploiting the deeply extracted features from some pre-trained deep models.

### Sample size impact

**Figure 2:**
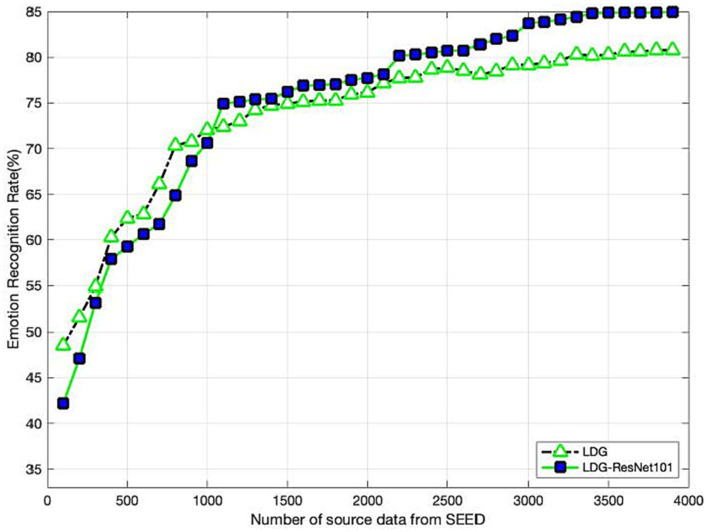
Recognition accuracy with varying sizes of source samples on SEED.

Figure 2 clearly reports the impact on the performance with different sizes of source on SEED, where the source size varies from 100 to 3,800. We can observe that our methods LDG and LDG + ResNet101 manifest the same trends of upgrade in the curves. As expected, larger source samples are beneficial to improve the recognition performance of our methods. It is worth noting that the performance of LDG can be smoothly and steadily improved with the increase of the source size, while LDG + ResNet101 can achieve significant performance when the source samples are relatively large, e.g., larger than 1,100. When the number of source samples increases to 3,500, LDG and LDG-ReNet101 asymptotically approach their equilibrium states.

### Multiple kernel selection

**Figure 3:**
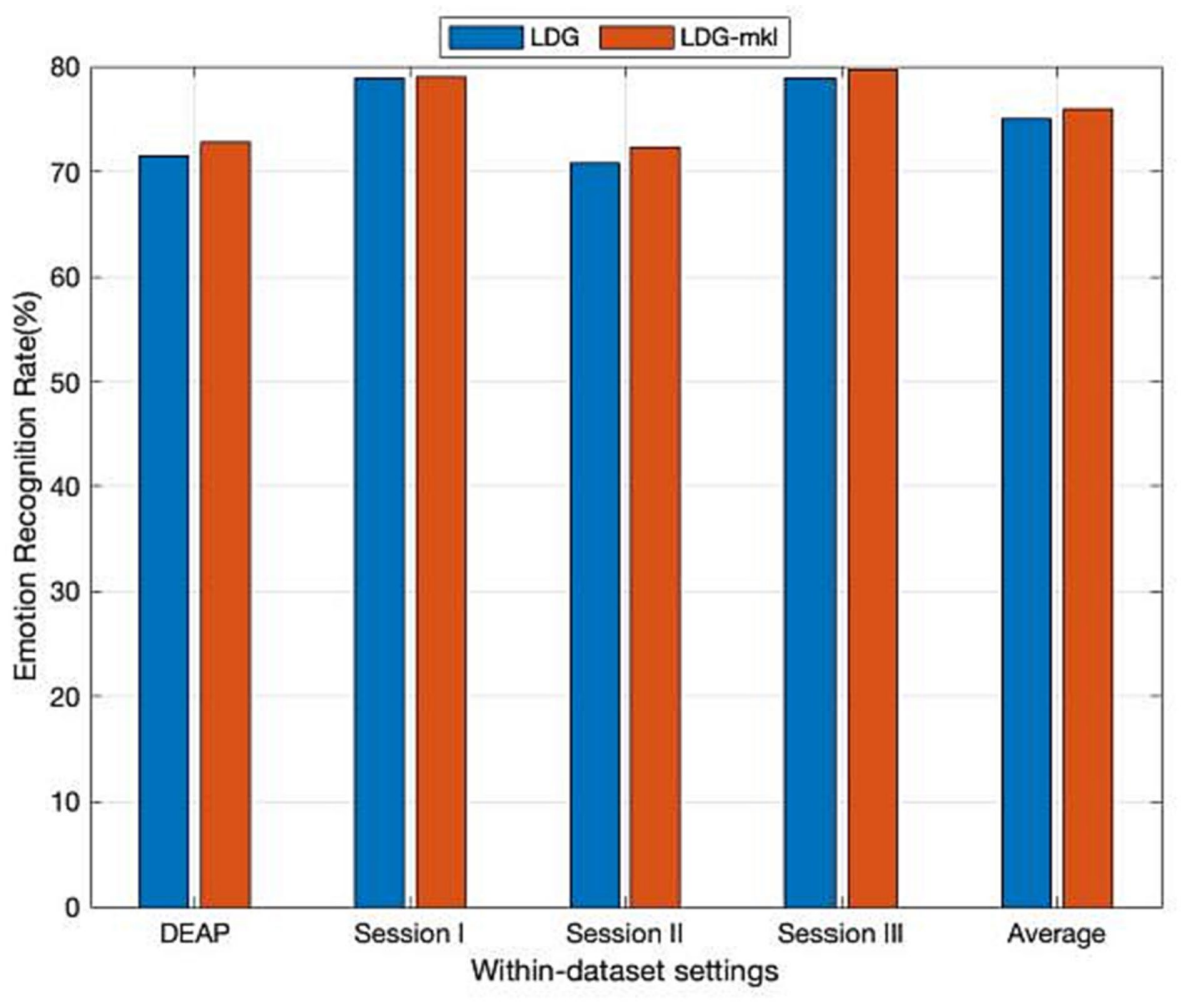
Domain adaptation emotion recognition on within-dataset with multi-kernel learning (SI: Session I, SII: Session II, SIII: Session III).

As an open problem, how to choose an effective kernel is a challenge for learning a kernel machine. Fortunately, the previously proposed multiple kernel learning (MKL) trick can be adapted to overcome this confusion. In the sequence, we further evaluate the performance improvement in our method via introducing MKL (denoted by LDG-mkl for short) for each subdomain, in which a new feature space spanned by multiple kernel projections will be first constructed. Specifically, given an empirical kernel function set ${\left\{\phi_{a}\right\}}_{a=1}^{℧}$, we, respectively, project them into ℧ different spaces, and then construct an orthogonally integrated feature space by horizontally concatenating these spaces. In this concatenated space, the projected features can be denoted by $\tilde{\phi }\left(x_{i}\right)={\left[\phi_{1}{\left(x_{i}\right)}^{T},\phi_{2}{\left(x_{i}\right)}^{T},\ldots ,\phi_{℧}{\left(x_{i}\right)}^{T}\right]}^{T}\in {\mathbb{R}}^{℧n_{a}}$, where $x_{i}\in X_{a}$, and then the kernel matrix can be easily deduced as $K_{new}=\left[{\tilde{K}}_{1};{\tilde{K}}_{2};\ldots ;{\tilde{K}}_{℧}\right]$, where ${\tilde{K}}_{i}$ is the *i*-th kernel matrix from the ℧ feature spaces. Following the same strategy in Long et al. (2015), besides the above-used Gaussian kernel, we additionally introduce another three kernel functions including inverse square distance kernel function, Laplacian kernel function, and inverse distance kernel function, which are, respectively, denoted as $K_{ij}=1/\left(1+\sigma {\left\|x_{i}-x_{j}\right\|}^{2}\right)$, $K_{ij}=\exp\left(-\sqrt{\sigma }\left\|x_{i}-x_{j}\right\|\right)$, and $K_{ij}=1/\left(1+\sqrt{\sigma }\left\|x_{i}-x_{j}\right\|\right)$. The observed mean experimental results from Figure 3 prove that LDG-mkl can boost the performance of LDG with a single kernel. This also verifies that the performance improvement in the kernel machines can be attributed to the diversities of multiple kernel functions.

### Cross-dataset domain generalization

In this subsection, we further evaluate the broad and consistent generalization capacity of our LDG method on cross-dataset emotion recognition. Intuitively speaking, cross-data generalization must be more challenging than cross-subject generalization due to the significant difference between datasets.

Following the same settings in Tao and Dan (2021) and Tao et al. (2022), for robust cross-dataset generalization, the 32 shared channels by SEED and DEAP are employed to support a common feature space of 160 dimensions. In this case, several cross-dataset generalization settings can be made up, i.e., DEAP→SI, DEAP→SII, DEAP→SIII, SI→DEAP, SEEDII→DEAP, and SIII→DEAP, where “*x* → *y*” means domain generalization from the dataset *x* into the dataset *y*, and SI, SII, and SIII are, respectively, denoted as the Session I, Session II, and Session III from SEED. When DEAP is regarded as the source dataset, 2,520 data are sampled from DEAP and 2,775 data taken as the target datasets are, respectively, sampled from three different sessions (SI, SII, and SIII) of SEED. When each session of SEED is taken as the source dataset, we resample 41,625 data from it as a training set and 180 samples from DEAP regarded as the target dataset. We report the mean generalization results on six cross-dataset in Table 3.

**Table 3:** Domain adaptation emotion recognition on cross-dataset.

Methods		DEAP → SI	DEAP → SII	DEAP → SIII	SI → DEAP	SII → DEAP	SIII → DEAP
Shallow methods	Undo-Bias	44.35	49.72	43.71	42.57	41.99	42.51
	UML	45.63	49.98	49.67	44.91	42.48	43.53
	DICA	47.35	52.68	52.11	43.34	44.90	42.46
	LRE-SVM	50.48	56.46	57.11	46.34	47.20	47.46
	SCA	48.89	54.35	54.65	**46.73**	45.36	44.67
	LDG	**52.62**	**57.66**	**57.83**	45.60	**47.89**	**49.76**
Deep methods	DDG	62.40	64.92	73.92	64.29	54.29	53.33
	DDC	60.89	62.43	69.43	62.16	52.16	50.07
	DANN	61.08	62.51	72.51	63.77	53.77	52.62
	DSAN	63.28	64.50	**74.50**	64.58	55.58	54.10
	CORAL	60.15	60.42	70.42	61.54	52.54	51.00
	CAN	64.22	65.77	75.77	66.12	**57.12**	55.39
	LDG+ Resnet101	**62.62**	**65.81**	74.42	**66.86**	55.68	**56.18**

Bold denotes the best recognition rates.

It can be seen from the experimental results in Table 3 that the average performance of each method on the cross-dataset is slightly worse than that in the within-dataset. This confirms that the distribution difference between the two datasets is greater than that between the two subjects. The superiority of subdomain generalization will be reflected in this scenario because subdomains can potentially alleviate the distribution diversity in cross-datasets when the target dataset is unavailable in the phase of training. This can also be proved by the observation from Table 3, where our method LDG outperforms other shallow methods in almost all cases. Although SCA occasionally achieves the best performance in one setting (SI → DEAP), our LDG method still achieves the top-one performance in other cases. In deep learning scenarios, all methods still undoubtedly outperform their shallow counterparts, which can be attributed to the advantage of deep feature representations. It is worth noting that our deep method LDG + Resnet101 also obtains the best or comparable recognition performance compared with other deep adaptation models. This once again proves the importance of the classification-level constraint in LDG.

Regarding the previously reported results in Yang et al. (2007), Tommasi et al. (2014), Tao et al. (2017, 2019, 2022), Ding et al. (2018a,b,c), and Tao and Dan (2021), multi-dataset adaptation can be improved by ensemble multiple auxiliary datasets. Please note that the scalability challenge could be incurred in case of multi-dataset generalization in that multi-dataset learning could bring the so-called “negative transfer” problem (Rosenstein et al., 2005), an open issue that exists in vanilla multi-source DA (Li J. et al., 2019; Chen et al., 2021; Tao and Dan, 2021). Therefore, we particularly evaluate the scalability of the proposed method by leveraging multiple source datasets for cross-dataset generalization. We report the average performance in Table 4 on all source datasets for the single-source methods including our LDG as well as LRE-SVM, Undo-Bias, and UML.

**Table 4:** Multi-dataset generalization (SI: Session I, SII: Session II, SIII: Session III).

Methods	{DEAP,SII,SIII} → SI	{DEAP,SI,SIII} → SII	{DEAP,SI,SII} → SIII	{SI,SII,SIII} → DEAP	{SI,SII} → DEAP	{SI,SIII} → DEAP
Undo-Bias	46.16	51.32	45.11	43.84	40.68	41.76
UML	44.06	50.10	51.21	46.01	42.90	43.85
DICA	49.28	52.94	54.06	46.62	45.39	44.63
LRE-SVM	47.17	57.30	59.30	**50.77**	46.50	49.06
SCA	52.33	**57.66**	57.29	48.68	**47.72**	48.93
LDG	**52.76**	**57.66**	**61.43**	49.34	47.03	**49.48**

Bold denotes the best recognition rates.

As shown in Table 4, due to the significant distribution differences among different source datasets, it is difficult for the single-source methods to generalize to unseen target domains in multi-source datasets. Therefore, the results in Table 4 indicate that these methods are generally inferior to other multi-source fusion methods. In some scenarios, they even exhibit a performance degradation trend as the number of source domains increases, indicating the “negative transfer” phenomenon (Rosenstein et al., 2005). Another interesting observation in Table 4 is that all multi-source methods achieve slight improvements by utilizing multiple sources as opposed to bridging only a single source (i.e., cross-dataset settings) as the number of source domains increases. This demonstrates the benefits of using multiple sources to enhance identification performance. In addition, SCA and DICA outperform other methods by conquering top-level performance as their designed weights are used to differentially screen the best sources. In some cases, our LDG method achieves more benefits than SCA. One possible explanation is that the discriminative information shared among sub-domain models in LDG is advantageous for multi-source generalization.

### Convergence

**Figure 4:**
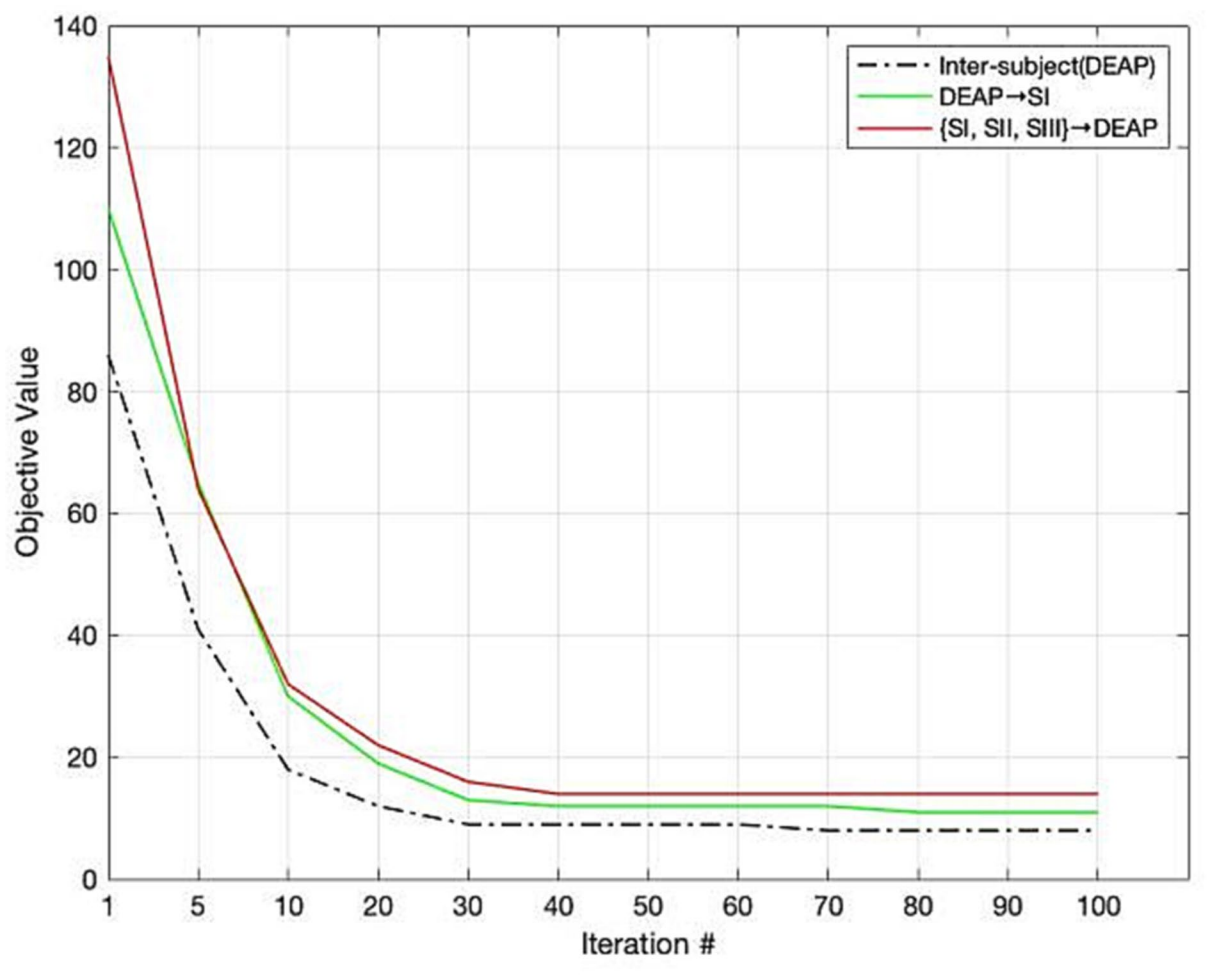
Convergence of LDG.

Since our LDG is an iterative algorithm, it is crucial to evaluate its convergence. We evaluate the convergence of the LDG algorithm by conducting several experiments for emotion recognition in three settings such as cross-subject within DEAP, DEAP→SI, and {SI, SII, SIII} → DEAP. We plotted the mean experimental results in Figure 4. The curves in this figure show that the proposed algorithm has a certain asymptotic convergence. The objective values of LDG usually converge in less than 30 iterations. We also observed a similar phenomenon from other recognition tasks with different cross-subject/cross-dataset settings.

## Conclusion

To deal with cross-subject/dataset EEG-based emotion recognition tasks, we proposed a local domain generalization (LDG) framework. In multiple subdomain spaces, LDG aims at transferring local knowledge into target learning mainly by leveraging correlation knowledge among subdomain models via low-rank constraint on the local models, which discriminatively screens unimportant prior evidence in subdomains. The comprehensive experiments performed on two public datasets verify the effectiveness of LDG in dealing with cross-subject/dataset emotion recognition. In most scenarios, our LDG and LDG + Resnet101 obtain the best results or comparable performance concerning several representative baselines.

Since the implementation of the LDG algorithm needs an iterative optimization procedure, how to improve the efficiency of LDG and seek a more efficient algorithm would be an issue worthy of further study in our future research. The unreliable and misleading pseudo-label strategy may be another potential problem in our LDG. Consequently, our successive work would be to explore seamlessly incorporating target labels into the framework of LDG.

## Data availability statement

Publicly available datasets were analyzed in this study. This data can be found here: in our research, the datasets DEAP and SEED can be, respectively, accessed from http://epileptologie-bonn.de/cms/upload/workgroup/lehnertz/eegdata.html and http://bcmi.sjtu.edu.cn/~seed.

## Author contributions

DZ extensively conducted all experiments in the paper. All authors contributed to the article and approved the submitted version.

## Footnotes

^1^It is worthy to note that the feature mapping function ø_*v*_ (1≤ *v* ≤ *m*) with respect to each local domain can be completely different from each other.
